# First Medical and Surgical Report of the Boston City Hospital

**Published:** 1871-03

**Authors:** 


First Medical and Surgical Report of the Boston City Hospital; edited by J. Nelson Borland, M.D. and David W. Cheever,

M.D. Boston: Little, Brown & Co.; for sale by S. C. Griggs & Co., Chicago.

This is a large volume of 688 pages, comprising an introductory history and description of the City Hospital, and a number of valuable articles by the different members of the Hospital Staff. These articles are illustrated by ten fine lithographic plates and two photographs. The following is the table of contents: History and Description of the Hospital; Perinephritic Abscess, by Henry I. Bowditch, M.D.; Excisions of Joints, by David W. Cheever, M.D.; Cases of Pneumonia, by J. Nelson Borland, M.D.; Displacement of the Upper Jaw, by David W. Cheever, M.D.; Treatment of Acute Rheumatism, by John G. Blake, M.D.; Treatment of Skin Diseases, by Howard F. Daman, M.D.; Typhoid and Typhus Fevers, by J. B. Upham, M. D.; Reproduction of the Tibia, by David W. Cheever, M.D.; Ophthalmic Reports, by Henry W. Williams, M.D.; Aural Reports, by J. O. Green, M.D.; Encepholoid Tumor of Tonsil; Occlusion of the Vagina, by David W. Cheever, M.D.; Peri-Uterine Inflammation, by Alex. D. Sinclair, M.D.; Surgical Abstract, by D. W. Cheever, M.D.; General Medical and Surgical Tables. The publication of permanent, well-considered reports from the medical and surgical staff of some of the best hospitals in this country, has been commenced within the last few years, and marks an important era in our medical literature. We hope these reports will receive sufficient patronage to make the practice permanent and universal.

